# FGFR1-Frs2/3 Signalling Maintains Sensory Progenitors during Inner Ear Hair Cell Formation

**DOI:** 10.1371/journal.pgen.1004118

**Published:** 2014-01-23

**Authors:** Kazuya Ono, Tomoko Kita, Shigeru Sato, Paul O'Neill, Siu-Shan Mak, Marie Paschaki, Masataka Ito, Noriko Gotoh, Kiyoshi Kawakami, Yoshiki Sasai, Raj K. Ladher

**Affiliations:** 1 Sensory Development, RIKEN Center for Developmental Biology, Chuo-ku, Kobe, Japan; 2 Neurogenesis and Organogenesis, RIKEN Center for Developmental Biology, Chuo-ku, Kobe, Japan; 3 Division of Biology, Center for Molecular Medicine, Jichi Medical University, Shimotsuke, Tochigi, Japan; 4 Department of Anatomy, National Defense Medical College, Tokorozawa, Japan; 5 Division of Genetics, Institute of Medical Science, University of Tokyo, Minato-ku, Japan; The University of Hong Kong, Hong Kong

## Abstract

Inner ear mechanosensory hair cells transduce sound and balance information. Auditory hair cells emerge from a Sox2-positive sensory patch in the inner ear epithelium, which is progressively restricted during development. This restriction depends on the action of signaling molecules. Fibroblast growth factor (FGF) signalling is important during sensory specification: attenuation of Fgfr1 disrupts cochlear hair cell formation; however, the underlying mechanisms remain unknown. Here we report that in the absence of FGFR1 signaling, the expression of Sox2 within the sensory patch is not maintained. Despite the down-regulation of the prosensory domain markers, p27^Kip1^, Hey2, and Hes5, progenitors can still exit the cell cycle to form the zone of non-proliferating cells (ZNPC), however the number of cells that form sensory cells is reduced. Analysis of a mutant Fgfr1 allele, unable to bind to the adaptor protein, Frs2/3, indicates that Sox2 maintenance can be regulated by MAP kinase. We suggest that FGF signaling, through the activation of MAP kinase, is necessary for the maintenance of sensory progenitors and commits precursors to sensory cell differentiation in the mammalian cochlea.

## Author Summary

The ability of our brain to perceive sound depends on its conversion into electrical impulses within the cochlea of the inner ear. The cochlea has dedicated specialized cells, called inner ear hair cells, which register sound energy. Environmental effects, genetic disorders or just the passage of time can damage these cells, and the damage impairs our ability to hear. If we could understand how these cells develop, we might be able to exploit this knowledge to generate new hair cells. In this study we address an old problem: how do signals from the fibroblast growth factor (FGF) family control hair cell number? We used mice in which one of the receptors for FGF (Fgfr1) is mutated and found that the expression of a stem cell protein, Sox2 is not maintained. Sox2 generally acts to keep precursors in the cochlea in a pre-hair cell state. However, in mutant mice Sox2 expression is transient, diminishing the ability of precursors to commit to a hair cell fate. These findings suggest that it may be possible to amplify the number of hair cell progenitors in culture by tuning FGF activity, providing a route to replace damaged inner ear hair cells.

## Introduction

The mammalian cochlea transduces sound using a dedicated sensory organ, the organ of Corti, which comprises of a highly ordered array of mechanosensory hair cells (HCs) and their associated support cells (SCs). The arrangement of cochlear HCs, 3 rows of outer hair cells (OHCs) and one row of inner hair cells (IHCs), together with SCs results from a balance between specification, progenitor expansion and differentiation [1].

The first step in HC specification is the induction of a Sox2-positive territory known as the sensory patch. Sox2 is critical for neurosensory precursor formation in the inner ear [2]–[4] and is induced by Notch signalling through its ligand Jagged (Jag)1 [5]–[9]. BMP signalling [10] then specifies the prosensory domain, the immediate precursors of the HCs and SCs, from within this Sox2-positive sensory patch. At specification, the prosensory domain exits the cell cycle, expressing the cell cycle inhibitor p27^Kip1^ as well as other prosensory domain markers. Importantly, the prosensory domain first becomes post-mitotic at the apical end of the cochlea from E12.5, spreading basally until E14.5 [11], [12].

HCs and SCs are picked out from within the prosensory domain through Notch signalling from putative SCs, acting on Delta1 or Jag2 in potential HCs [5], [13]–[15]. This lateral inhibition ensures that only some of the cells of the prosensory domain retain the transcription factor Atoh1 [16], [17]. Atoh1 is both necessary and sufficient for HC differentiation [18]. In contrast to the apical to basal wave of cell cycle exit of the prosensory domain, the wave of differentiation occurs basally at E14.5 extending apically at E17.5 [19].

In addition to the above, fibroblast growth factor (FGF) signalling has also been shown to be important in the development of the cochlear HC. Conditional deletion of *Fgf receptor (Fgfr) 1*, results in the loss of HCs [20]. This phenotype is observed to a lesser extent, when the proposed ligand for FGFR1, *Fgf20*, is deleted [21]. *Ex vivo* explant studies suggest that FGF signalling enhances Notch-Jag signalling after sensory patch induction [22]. However the *in vivo* significance of these observations and how they lead to the *Fgfr1* deletion phenotype are not clear.

Fgf ligand binding causes the dimerization and activation of the canonical receptor tyrosine kinase [23]. Activation, generally by phosphorylation of particular tyrosine residues in the intracellular domain of the Fgf receptor, results in recruitment of adaptor proteins that are essential for the intracellular response to the extracellular signal. Each group of phosphorylated residues mediate distinct functions, for example phosphorylation of tyrosine 766 in FGFR1 serves as a potential binding site for phospholipase C-γ (PLCγ) [24]. Other adaptor proteins include Fgf Receptor Substrate (Frs) 2 or 3 (collectively termed Frs2/3) [25], [26]. Frs2/3 recruitment and activation leads to the stimulation of multiple FGFR-dependent signaling pathways such as Ras/MAP kinase pathway, and the phosphatidylinositol-3-kinase (PI3K) pathway [27]. Studies into a mouse allele in which the Frs2/3 interaction motif has been deleted, reveal that Frs2/3 recruitment mediates aspects of FGFR1 signalling [28]. However, the necessity of these pathways in inner ear development had not been investigated.

In this study, we found that FGFR1 signalling through Frs2/3 is necessary for prosensory formation. Even in the absence of FGFR1-Frs2/3 signalling, the prosensory domain becomes post-mitotic, however the expression of prosensory markers is impaired. This results in fewer sensory precursors, giving rise to a reduction in HC numbers. We also found that the expression of Sox2 is transient, suggesting that the strength and duration of Sox2 expression, under the direct or indirect control of FGF-mediated MAP kinase activation, commits progenitors to sensory cell differentiation.

## Results

### FGFR1 signalling through Frs2/3 recruitment is required for normal cochlear hair cell development

**Figure 1:**
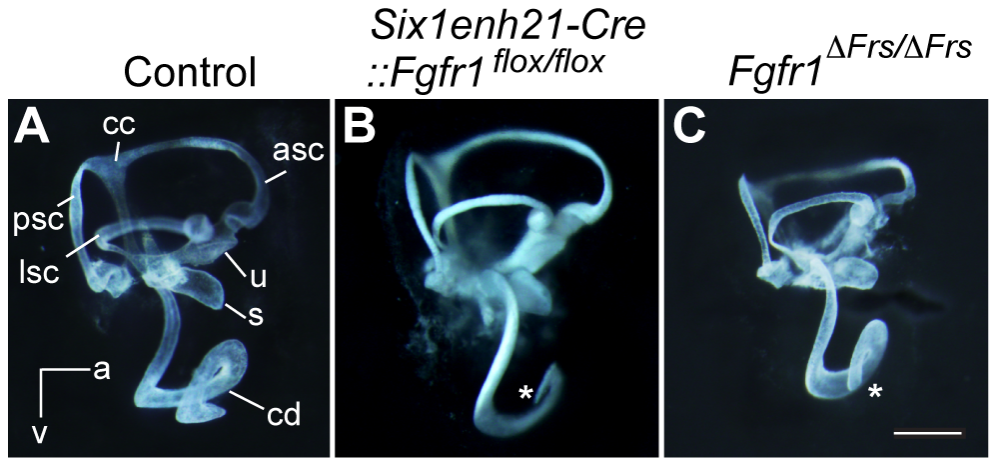
Inner ear development is disrupted in *Fgfr1* mutants. (A) The morphology of the inner ear was revealed by the injection of white paint. Control inner ears show a typical morphology consisting of 3 semi-circular canals and the spiral cochlear duct that is 1.5 turns long. (B) The inner ear of *Six1enh21-Cre:: Fgfr1^flox/flox^* exhibits only 1 turn of the cochlear duct (asterix). Vestibular components are unaffected. (C) *Fgfr1* ^Δ*Frs*/Δ*Frs*^ inner ear shows milder cochlear phenotype than that of *Six1enh21-Cre::Fgfr1^flox/flox^* with a slightly shortened cochlear duct (asterix). cd, cochlear duct; u, utricle; s, saccule; psc, posterior semicircular canal; asc, anterior semicircular canal; lsc, lateral semicircular canal; cc, common crus; a, anterior; v, ventral.

To determine gross morphology, the inner ear from *Six1enh21-Cre::Fgfr1^flox/flox^* and *Fgfr1* ^Δ*Frs/*Δ*Frs*^ at E14.5 were examined first by paint-filling [29]. The cochlear duct of the conditional mutant (*Six1enh21-Cre::Fgfr1^flox/flox^*) was shorter than control (Figure 1A and B). *Fgfr1* ^Δ*Frs/*Δ*Frs*^ also exhibited a truncated cochlear duct although the phenotype was milder than that of the conditional mutant (Figure 1C). No significant difference in the formation of vestibular components was observed.

**Figure 2:**
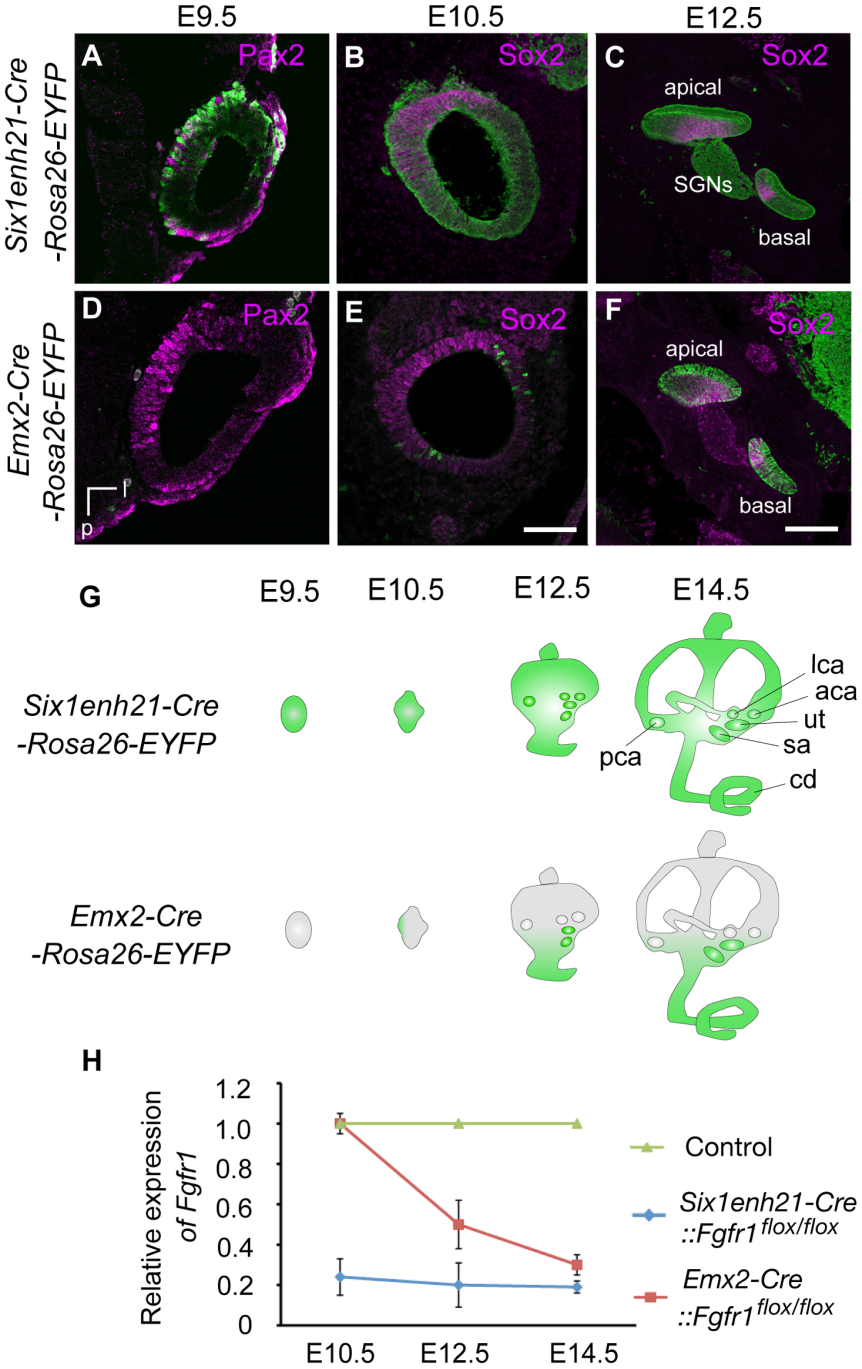
Spatio-temporal activity of conditional Cre drivers. (A–F) EYFP expression under control of *Six1enh21-Cre* or *Emx2-Cre* at E9.5, E10.5, and E12.5. Six1enh21-Cre-mediated EYFP expression is detectable in a majority of otic cells, marked by Pax2, by E9.5 (A). All of Sox2 expressing otic cells colocalize with Cre-mediated EYFP by E10.5 (B). The early activity results in detectable EYFP expression in the spiral ganglion cells as well as epithelial cells that include sensory competent cells marked by Sox2 at E12.5 (C). In contrast, very few Emx2-Cre-mediated EYFP expressing cells can be detected at E9.5 (D). The onset of Emx2-Cre activity in the inner ear is around E10.5 in the lateral wall of otocyst (E), and becomes uniform by E12.5 throughout the cochlear duct (F). (G) Schematic illustration depicting spatio-temporal activity (green) of each Cre driver during inner ear development. (H) RT-PCR analysis showing temporal deletion of *Fgfr1* by two Cre lines. Mean ± SD are shown. cd, cochlear duct; u, utricle; s, saccule; psc, posterior semicircular canal; asc, anterior semicircular canal; lsc, lateral semicircular canal; l, lateral; v, ventral; p, posterior; SGNs, spiral ganglion neurons. Scale bars: A, B, D, and E, 75 µm (in E); C and F, 150 µm (in F).

A requirement for FGFR1 function in cochlear HC development had been previously shown [20], however the mechanisms used remained unknown. We asked when FGFR1 signalling was acting during HC development, by exploiting the difference in the timing activation of of two different Cre driver lines. To first verify Cre activity, we crossed these lines with a *Rosa26-flox-STOP-flox-EYFP* reporter, in which the expression of EYFP is initiated after the Cre-mediated excision of the STOP, transcription terminator sequence. *Six1enh21-Cre* activity can be detected as early as E9.5 specifically in whole otic epithelium (Figure 2A–C and G). In contrast, *Emx2-Cre* activity cannot be detected at E9.5, but is active at E12.5, with EYFP labelled in the almost all putative sensory organs except three semicircular ampullae (Figure 2D–G and data not shown). Quantitative PCR for the deleted portion of *Fgfr1* confirmed the temporal activity of the two Cre lines (Figure 2H). *Fgfr1* levels in the cochlear rudiment were reduced to approximately 20% of normal in *Six1enh21-Cre::Fgfr1^flox/flox^* from E10.5. In contrast, *Fgfr1* levels in *Emx2-Cre::Fgfr1^flox/flox^* cochleae were close to wild-type levels at E10.5, falling to 60% at E12.5 and 20% by E14.5. We thus used these lines to examine the cochlear phenotypes when *Fgfr1* deletion occurred at around E9.5 to 10.5 (using *Six1enh21-Cre*) or at around E12.5 (using *Emx2-Cre*).

**Figure 3:**
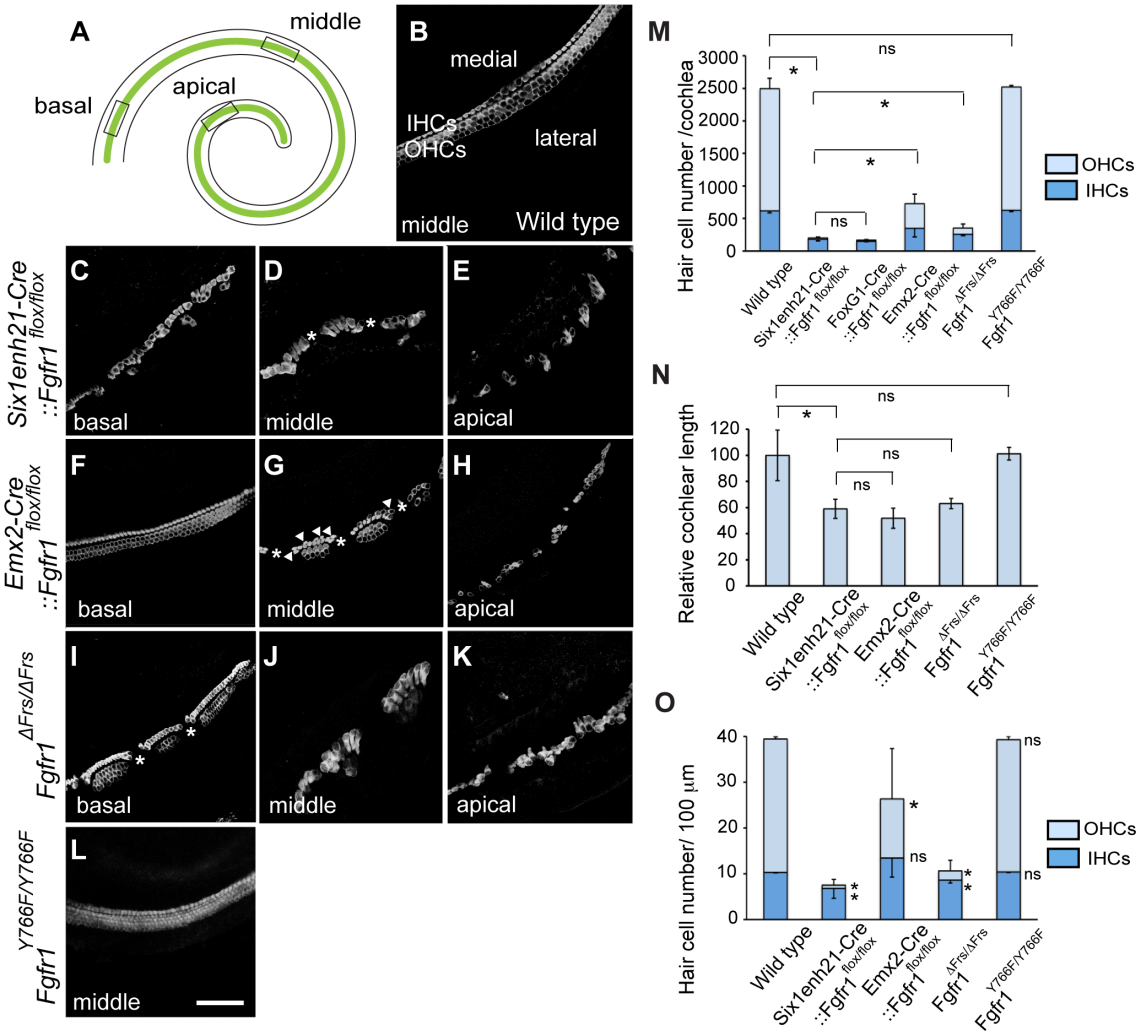
Cochlear hair cells are reduced in *Fgfr1* mutants. (A) Schematic of whole mount view of neonatal cochlear duct. HCs are arranged along the entire length of cochlear duct. (B) Magnified view of wild type cochlear duct labeled with anti-Myo7a antibody. Highly ordered array of three rows of OHCs and one row of IHCs are observed. (C–E) HCs are disrupted in *Six1enh21-Cre::Fgfr1^flox/flox^* cochlea. Basally, few OHCs are visible (C). In the middle, there are small gaps in the remaining IHCs (asterix) (D). Apically, sparsely distributed HCs are observed (E). (F–H) *Emx2-Cre::Fgfr1^flox/flox^* mice show a less severe phenotype. Basally, IHCs and OHCs form, although only two rows of OHCs form (F). In the middle region, islands of HCs form between gaps (asterix). Additional IHCs are occasionally observed (arrowheads) (G). Apically, islands are sparse (H). (I–K) *Fgfr1* ^Δ*Frs/*Δ*Frs*^ cochlea shows a less severe phenotype than that of *Six1enh21-Cre::Fgfr1^flox/flox^* cochlea, but more severe phenotype than *Emx2-Cre::Fgfr1^flox/flox^* cochlea. Basally, some IHCs and OHCs form, although frequently arranged in islands, punctuated by gaps (asterix). (L) The number of rows of HCs and their patterning are unaffected in *Fgfr1^Y766F/Y766F^* mutant cochlea. (M) Stacked graph comparing the number of cochlear IHCs and OHCs amongst different *Fgfr1* mutants. Sum of each number represents total HC number. Statistic significance is shown for total HC number. Error bars (mean ± SD) represent variation in each HC type. (N) Graph showing relative length of cochlear duct at E18.5. Cochlear length is decreased by approximately 40–50% in all *Fgfr1* mutants except *Fgfr1^Y766F/Y766F^*. (O) Number of IHCs and OHCs are counted and normalized to 100 µm from different *Fgfr1* mutants. Sum of each number represents total HC number per 100 µm. Statistic significance is shown for each HC type, compared with the number of HCs from wild type. Error bars (mean ± SD) represent variation in each HC type. *p<0.05. ns, not significant. Scale bar: C–L; 50 µm (in L).

To investigate HC phenotype, whole-mount cochlear samples from E18.5 mice were dissected and immunostained for Myo7a. Control, wild-type, cochleae showed the typical arrangement of three rows of OHCs and one row of IHCs along the entire length of the cochlea (Figure 3A, B). In *Six1enh21-Cre::Fgfr1^flox/flox^* the arrangement of HCs was altered, with those in the apical third of the cochlea more severly affected (Figure 3C–E). Here the rows of HCs were discontinuous, and arranged in islands. Typically, OHCs were missing, although isolated OHCs could be found basally. The cochlear phenotype of *Emx2-Cre::Fgfr1^flox/flox^* inner ears was milder (Figure 3F–H). Basally, OHC loss was less pronounced with the outer-most row most severely affected (Figure 3F). Further apically, the HC row became discontinuous, and islands that were present were made up of IHCs and OHCs, with occasional additional IHCs observed (Figure 3G). HCs were more sparsely distributed in the apical-most part of the cochlea (Figure 3H).

We next addressed the downstream pathway employed by FGFR1 during cochlear HC formation using two alleles of *Fgfr1*, *Fgfr1* ^Δ*Frs/*Δ*Frs*^ and *Fgfr1^Y766F/Y766F^*. Y766F carries a point mutation converting a tyrosine at position 766 to a phenylalanine, rendering it resistant to phosphorylation. This has been postulated to result in a failure of PLCγ phosphorylation and thus its activation [30]. The cochlear HC phenotype of *Fgfr1* ^Δ*Frs/*Δ*Frs*^ inner ears closely resembled that of *Six1enh21-Cre::Fgfr1^flox/flox^*, showing the severe OHC loss apically and the islands of HCs (Figure 3I–K). In contrast, surface preparations from the inner ear of *Fgfr1^Y766F/Y766F^* showed that cochlear HCs were normal (Figure 3L). The correspondance of the HC phenotypes was confirmed after quantifying the number of cochlear HCs, and also compared to the previously published *Foxg1-Cre::Fgfr1^flox/flox^* [20]. The total number of HCs per cochlea averaged 2494±160 (n = 4) in wild-type controls. There were 201±26 (n = 4) HCs in *Six1enh21-Cre::Fgfr1^flox/flox^* inner ears, 218±44 in *Foxg1-Cre::Fgfr1^flox/flox^* cochlea (n = 4), 728±274 (n = 6) in *Emx2-Cre::Fgfr1^flox/flox^*, 420±60 (n = 5) in *Fgfr1* ^Δ*Frs/*Δ*Frs*^, and 2532±23 (n = 6) in *Fgfr1^Y766F/Y766F^* (mean ± SD, respectively) (Figure 3M). The significant difference was also determined when comparing *Six1enh21-Cre::Fgfr1^flox/flox^* and *Emx2-Cre::Fgfr1^flox/flox^* cochleae (*p*<0.05). Given the differences in the timing of the two Cre drivers (Figure 2), these results suggest that FGFR1 signalling commences prior to E12.5.

Next we counted the number of IHCs and OHCs (Figure 3M). By comparisons with control cochlea (1876±160), OHC loss were evident in *Six1enh21-Cre::Fgfr1^flox/flox^* (22±14, decreased by 99%), *Emx2-Cre::Fgfr1^flox/flox^* (379±144, decreased by 80%), and *Fgfr1* ^Δ*Frs/*Δ*Frs*^ (93±77, decreased by 95%), but not in *Fgfr1^Y766F/Y766F^* (1894±24). With the exception of *Fgfr1^Y766F/Y766F^* mutants (626±19), the number of IHCs were also reduced in FGFR1 signaling mutants; *Six1enh21-Cre::Fgfr1^flox/flox^* (179±25, decreased by 72%), *Emx2-Cre::Fgfr1^flox/flox^* (349±130, decreased by 44%), and *Fgfr1* ^Δ*Frs/*Δ*Frs*^ (259±61, decreased by 58%), compared with wild type control (618±31) (*p*<0.05). In addition, cochlear length was decreased by 41% in *Six1enh21-Cre:: Fgfr1^flox/flox^*, by 49% in *Emx2-Cre::Fgfr1^flox/flox^*, and by 37% in *Fgfr1* ^Δ*Frs/*Δ*Frs*^ mutants, respectively (Figure 3N). To exclude the influence of cochlear length on total HC number, we counted the number of each HC type normalized to 100 µm length (Figure 3O). IHCs were decreased (*p*<0.05) by 33% in *Six1enh21-Cre::Fgfr1^flox/flox^* (6.8±2.1), by 17% in *Fgfr1* ^Δ*Frs/*Δ*Frs*^ (8±0.1) of wild type levels (10.3±0.1). However, normalized number of IHCs was statistically the same in *Emx2-Cre::Fgfr1^flox/flox^* (13.4±4.1) and wild type (10.3±0.1). In contrast, OHC number per 100 µm was decreased by 98% in *Six1enh21-Cre::Fgfr1^flox/flox^* (0.7±1.2), by 56% in *Emx2-Cre::Fgfr1^flox/flox^* (12.8±11.1), and by 87% in *Fgfr1* ^Δ*Frs/*Δ*Frs*^ (2.0±2.2) when compared to wild type levels (29.1±0.4). These findings suggested that FGFR1-Frs2/3 activity was required for OHC development from E10.5, whereas FGFR1-Frs2/3 activity was only required for IHC development prior to E12.5. Taken together, these results demonstrate that signalling via Frs2/3 recruitment is necessary for FGFR1 activity during the formation of the cochlear HCs.

**Figure 4:**
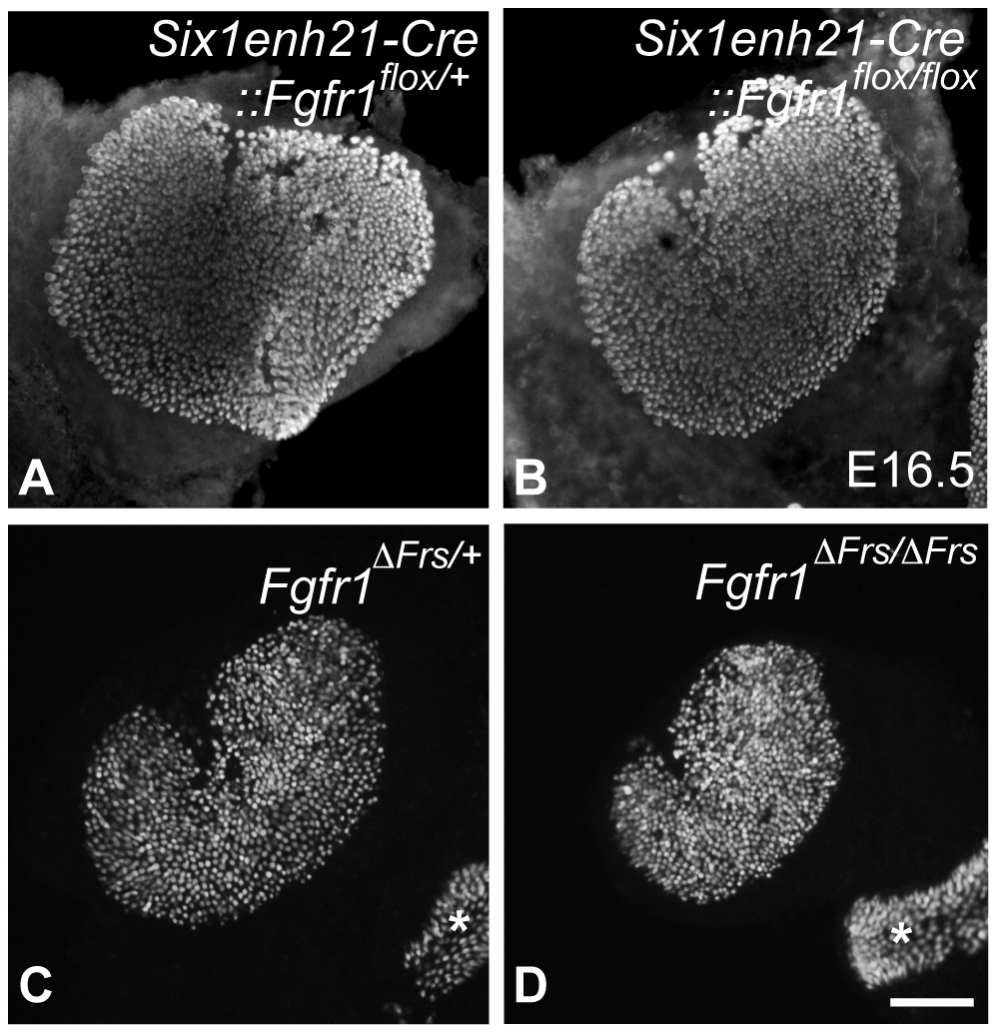
Disruption of FGFR1-Frs2/3 pathway decreases the number of vestibular hair cells. (A, B) No change in HC number was detected between E16.5 *Six1enh21-Cre::Fgfr1^flox/+^* (A) and *Six1enh21-Cre::Fgfr1^flox/flox^* utricles (B) labeled with anti-Myo7a antibody. (C, D) Fewer HCs were observed in the utricle of E16.5 *Fgfr1* ^Δ*Frs/*Δ*Frs*^ mutants (D) than *Fgfr1* ^Δ*Frs/+*^ control (C). Ampullary HCs are also included (asterisks). Scale bar: A–D; 200 µm (in D).

In addition to the cochlear HC phenotype, we analyzed the number of HCs in one of the vestibular sense organs, the utricle dissected from E16.5 mice. While utricilar HCs number was comparable between *Six1enh21-Cre:: Fgfr1^flox/+^* (577±21, n = 3) and *Six1enh21-Cre:: Fgfr1^flox/flox^* (550±10, n = 4) (Figure 4A, B), HC number was significantly decreased (*p*<0.05) in *Fgfr1* ^Δ*Frs/*Δ*Frs*^ mutants (473±57, n = 6), by comparison with *Fgfr1* ^Δ*Frs/+*^ control (718±81, n = 4) (Figure 4C, D). As this mutant is non-conditional, it may suggest that FGFR1 signalling outside of the inner ear epithelium plays a role in vestibular HC formation.

### Support cell development is perturbed in the absence of Frs2/3-mediated FGFR1 signalling

**Figure 5:**
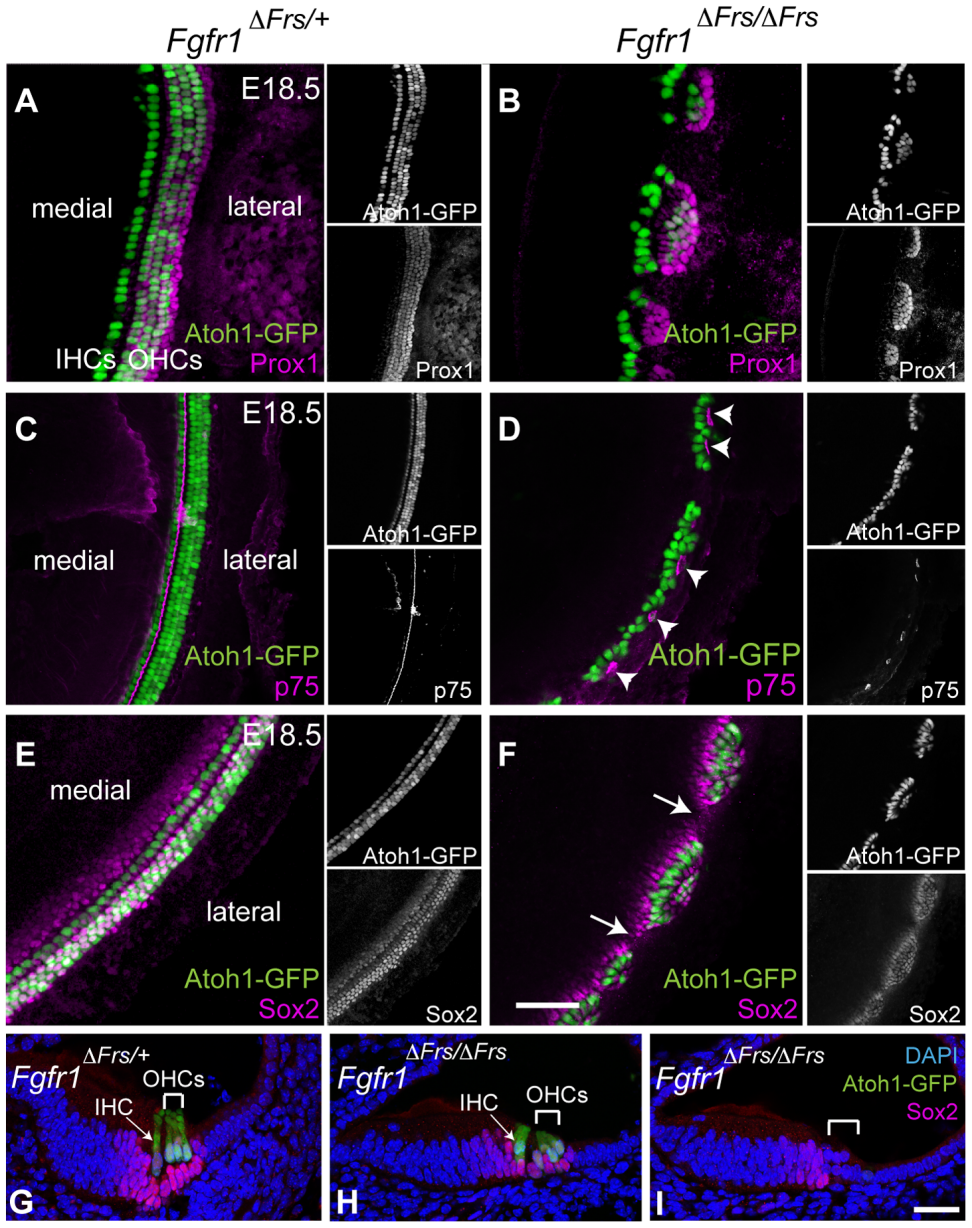
Disruption of FGFR1-Frs2/3 pathway decreases the number of support cells. (A) Prox1 immunostaining (magenta) marks cochlear SCs; the pillar cells and Deiter's cells in heterozygous *Fgfr1* ^Δ*Frs/+*^ cochlea at E18.5. This line also expresses GFP under the control of the Atoh1 enhancer marking HCs (green). (B) In mutant *Fgfr1* ^Δ*Frs/*Δ*Frs*^ cochlea, HCs form Atoh1-GFP-positive sensory islands. Prox1-positive SCs are not detected in the space between these islands. (C) In *Fgfr1* ^Δ*Frs/+*^ control cochlea at E18.5, expression of p75 (magenta), a marker for pillar cells, marks adjacent to the IHCs. (D) p75 is observed in patches in mutant *Fgfr1* ^Δ*Frs/*Δ*Frs*^ cochlea (arrowheads), and it is not detected in the gaps between islands. Note that most Atoh1-GFP-positive HCs are located medial to p75-expressing pillar cells. (E) In heterozygous *Fgfr1* ^Δ*Frs/+*^ cochlea at E18.5, Sox2 (magenta) marks SCs arranged in rows coincident with Atoh1-GFP-positive HCs. (F) In mutant *Fgfr1* ^Δ*Frs/*Δ*Frs*^ cochlea, Sox2 expression extends beyond the sensory islands, but is not detected in the gaps (arrows). (G) Transverse section view of organ of Corti from E18.5 *Fgfr1* ^Δ*Frs/+*^ expressing GFP under the control of the Atoh1 enhancer, labeled with Sox2. One IHC (arrow) and three OHCs (bracket) are observed. Sox2 is expressed in surrounding SCs, including Deiter's cells, pillar cells, inner phalangeal cell, and Kölliker's organ. (H) Cross section of a sensory island from E18.5 mutant *Fgfr1* ^Δ*Frs/*Δ*Frs*^ cochlea. Here, one IHC (arrow) and two OHCs (bracket) are detected. Sox2 expression is detected in surrounding SCs. (I) Cross section of a gap intervening sensory islands from mutant *Fgfr1* ^Δ*Frs/*Δ*Frs*^ cochlea. No HCs are detected and Sox2 is only detectable in Kölliker's organ, but not in lateral compartment (bracket). Nuclei are visualized by DAPI. Scale bars: A–F, 50 µm in (in F); G–I, 30 µm (in I).

The decision by sensory precursors to generate either HCs or SCs is controlled by Notch-Delta cell-cell signalling [5], [13]–[15]. Therefore, one possible mechanism of FGFR1 action is in modifying the action of Notch and Delta in this choice. We thus investigated whether SC formation was disrupted in the absence of Frs2/3-mediated FGFR1 signalling. We crossed *Fgfr1* ^Δ*Frs/*Δ*Frs*^ onto an *Atoh1-GFP* reporter line to reveal HCs. At E18.5, Prox1 is strongly expressed in the Deiter's cells and in the pillar cells [31]. In the control, *Fgfr1* ^Δ*Frs/+*^ cochlea, Prox1-labeled 5 rows of cells (Figure 5A). In mutant *Fgfr1* ^Δ*Frs/*Δ*Frs*^ cochlea, only two to three rows of Prox1-labelled cells were detected and were confined within sensory islands (Figure 5B). In whole mount view of *Fgfr1* ^Δ*Frs/+*^ cochlea, p75 expression was apparent in the inner pillar cells that are found along the length of the cochlear duct (Figure 5C). In *Fgfr1* ^Δ*Frs/*Δ*Frs*^ cochlea, p75 staining was only found in the sensory cell islands highlighted by Atoh1-GFP and not found in the intervening spaces (Figure 5D). Within severely affected region, the row of p75-positive cells was mostly present lateral to the one row of HCs, suggesting that these islands were exclusively IHCs.

The other SC marker at this stage, Sox2, was also only found within the sensory islands (Figure 5E and F). Section analysis revealed that Sox2 is expressed in SCs, in both control organ of Corti (Figure 5G) and in sections taken through the level of the islands in *Fgfr1* ^Δ*Frs/*Δ*Frs*^ cochlea (Figure 5H). In sections taken through the gaps in between the islands, we could only detect weak Sox2 expression in the Kölliker's organ, a region medial to lateral compartment (Figure 5I). Combined, these results suggest that the FGFR1-Frs2/3 signalling axis also affects the formation of SCs, and is thus acting upstream of HC/SC decision mediated by Notch-Delta signalling.

### FGFR1/Frs2/3 interaction is not essential for FGFR1-mediated cell cycle regulation during inner ear development

Precursors of auditory HCs and SCs form from a domain known as the prosensory domain [1]. This region emerges from within the Sox2-positive sensory patch between E12.5 and E14.5, depending on the exact position within the cochlea. It is initially characterised by the cessation of mitosis, forming the zone of non-proliferating cells (ZNPC), as well as the expression of a cell cycle inhibitor, p27^Kip1^. Subsequently, the ZNPC expresses specific markers of the prosensory domain such as Hey2 and Hes5. It had been previously shown that a conditional deletion of *Fgfr1* regulates proliferation in the cochlea [20]. We thus asked if cell cycle regulation within the cochlea was mediated by FGFR1-mediated Frs2/3 activity.

**Figure 6:**
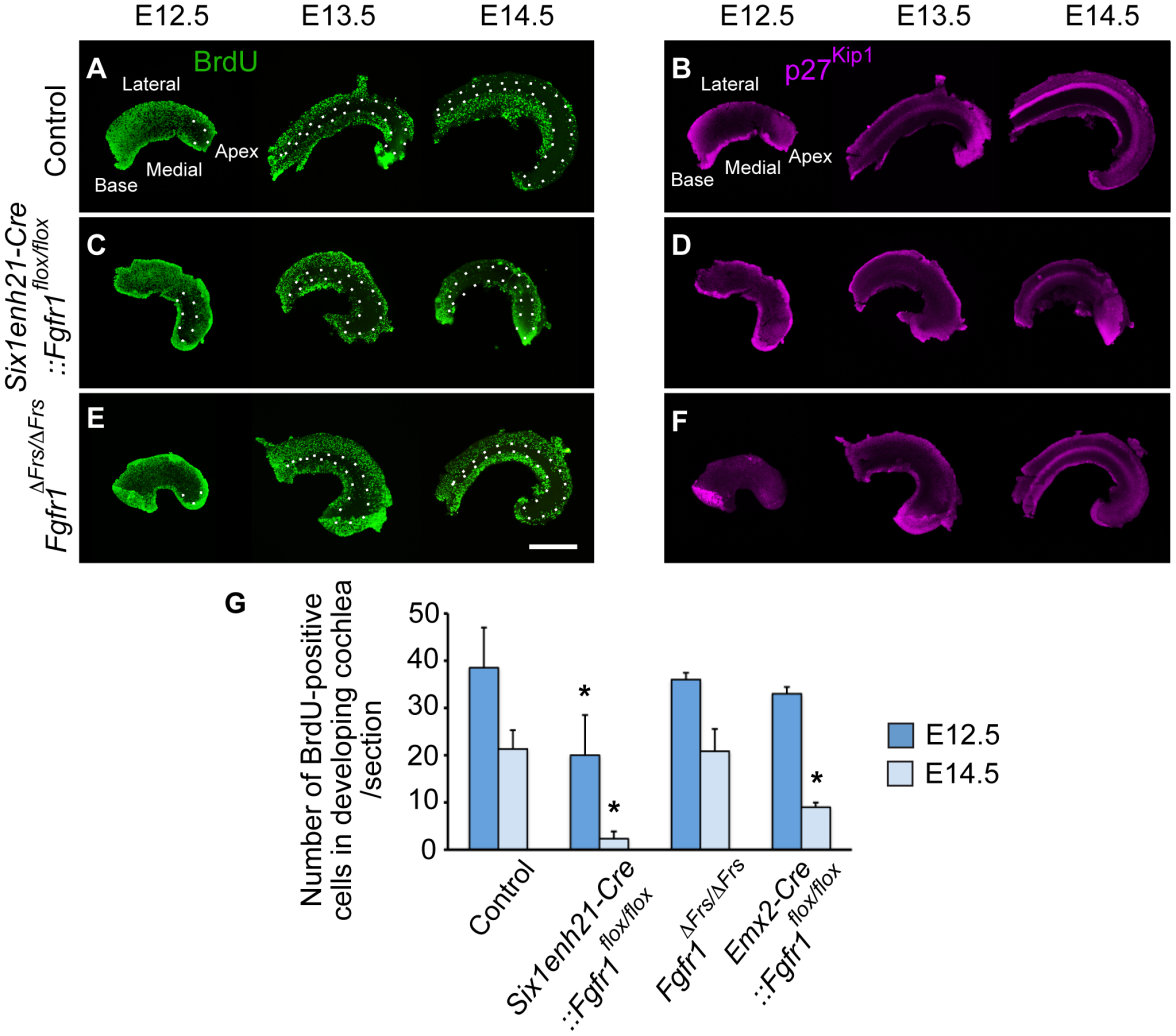
FGFR1-mediated cell proliferation is not mediated by Frs2/3 interaction. Cochlear whole mounts from controls (A, B), *Six1enh21-Cre::Fgfr1^flox/flox^* (C, D) and *Fgfr1* ^Δ*Frs/*Δ*Frs*^ (E, F) were assessed for incorporation of BrdU injected 2 hours before sacrifice (A, C, E) and the expression of p27^Kip1^ (B, D, F) at E12.5, E13.5, and E14.5. (A) The pattern of BrdU uptake indicates that the ZNPC (circled by dotted line) is first apparent at E12.5 in the apex of control cochlea and extends basally by E14.5 in control cochlea. At E13.5 and E14.5, proliferation in Kölliker's organ medial to the ZNPC is apparent. (B) p27^Kip1^ protein expression is co-incident with the ZNPC in control cochlea at E12.5 to E14.5. (C) The ZNPC was still detected in *Six1enh21-Cre::Fgfr1^flox/flox^* cochlea apically at E12.5, extending basally by E14.5. Reduced BrdU uptake was noted in Kölliker's organ. (D) p27^Kip1^ protein expression in apical region was normal at E12.5, but reduced in *Six1enh21-Cre::Fgfr1^flox/flox^* cochlea at E13.5 and E14.5. (E) BrdU immunostaining revealed that the ZNPC also formed normally in *Fgfr1* ^Δ*Frs/*Δ*Frs*^ cochlea from E12.5. However, robust proliferation was noted in Kölliker's organ at E13.5 and E14.5. (F) p27^Kip1^ protein expression in apical region was normal at E12.5, but reduced in *Fgfr1* ^Δ*Frs/*Δ*Frs*^ cochlea at E13.5 and E14.5, compared to control. (G) Graph showing the number of BrdU-positive cells at in the cochlea at E12.5 and in Kölliker's organ at E14.5. BrdU-labelled cells were reduced in *Six1enh21-Cre:: Fgfr1^flox/flox^* cochleae at both E12.5 and E14.5. In *Emx2-Cre::Fgfr1^flox/flox^* mutant cochlea, there was no significant reduction in proliferation at E12.5, but a reduction in BrdU uptake was noted in Kölliker's organ at E14.5. No difference was detected between control and *Fgfr1* ^Δ*Frs/*Δ*Frs*^ cochlea. *p<0.05. Scale bar: 75 µm.

We first asked if *Six1enh21-Cre:: Fgfr1^flox/flox^* mutants used in this study recapitulated the reported cell cycle deficit shown previously in *FoxG1-Cre:: Fgfr1^flox/flox^* [20]. Prosensory domain progenitors become post-mitotic commencing at the apex at E12.5 and ending at the base at E14.5. Cell cycle exit correlates with the expression of p27^Kip1^, as was observed in whole mount preparations of control heterozygous cochlea stained for p27^Kip1^ and BrdU (Figure 6A and B). Consistent with previous observations, no cell cycle defect was detected in *Six1enh21-Cre:: Fgfr1^flox/flox^* mutant at E10.5 (data not shown) [20]. However, a reduction in cell proliferation within the epithelial cells of the cochlea was detected in *Six1enh21-Cre:: Fgfr1^flox/flox^* mice at E12.5 (Figure 6C). This phenotype was more prominent in Kölliker's organ at E13.5 and E14.5. Surprisingly, and despite the proper formation of the ZNPC, p27^Kip1^ was down-regulated in *Six1enh21-Cre:: Fgfr1^flox/flox^* cochleae at E13.5 and E14.5 when compared to controls (Figure 6D). Quantification of BrdU-labelled cells showed far fewer proliferating cells in E12.5 nascent cochlear duct of *Six1enh21-Cre:: Fgfr1^flox/flox^* (14±8: n = 5 compared with 41±8: n = 4 in wild type controls) and E14.5 Kölliker's organ (2±2: n = 5 compared with 21±4: n = 5 in wild type controls) (*p*<0.05) (Figure 6G).

We next investigated proliferation in *Fgfr1* ^Δ*Frs/*Δ*Frs*^ cochlea. *Fgfr1* ^Δ*Frs/*Δ*Frs*^ mutant cochleae still exhibited down-regulation of p27^Kip1^ throughout cochlear duct (Figure 6F). However in contrast to *Six1enh21-Cre:: Fgfr1^flox/flox^* mutant cochlea, BrdU-positive cells were observed in Kölliker's organ of *Fgfr1* ^Δ*Frs/*Δ*Frs*^ mutant (Figure 6E). Comparable number of BrdU-positive cells were detected in *Fgfr1* ^Δ*Frs/*Δ*Frs*^ at both stages (36±8: n = 4 at E12.5, and 21±5: n = 5 at E14.5) (*p*<0.05) (Figure 6G). We also quantified the number of BrdU-positive cells in *Emx2-Cre::Fgfr1^flox/flox^* cohleae. Reduced proliferation was only detected at E14.5 and was milder than that observed for *Six1enh21-Cre:: Fgfr1^flox/flox^* (39±1: n = 4 at E12.5, and 9±1: n = 4 at E14.5). These results indicate that Frs2/3 recruitment does not mediate FGFR1-induced cell proliferation in Kölliker's organ during inner ear development. Furthermore, these results showed that FGFR1-Frs2/3 signaling is not necessary for the formation of the ZNPC, but is required for p27^Kip1^ expression.

### Formation of the prosensory domain is disrupted by lack of FGFR1-Frs2/3 signalling

**Figure 7:**
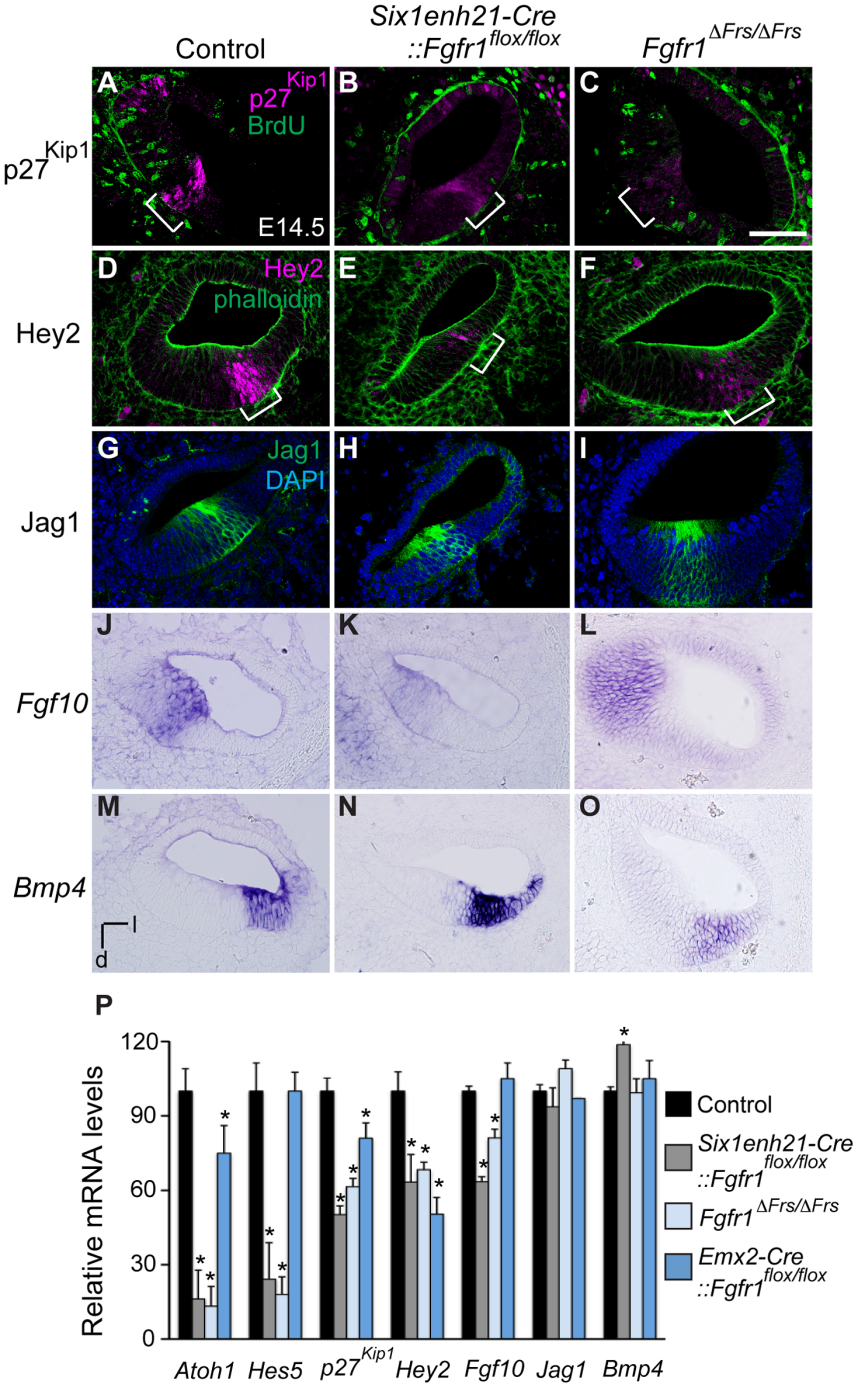
FGFR1-Frs2/3 pathway is required for the specification of prosensory domain. (A–C) p27^Kip1^ (magenta) is expressed in the post-mitotic, BrdU (green) negative, prosensory domain (bracket) in sections of E14.5 cochlea (A). Expression of p27^Kip1^ is decreased in *Six1enh21-Cre:: Fgfr1^flox/flox^* cochlea (B) and in *Fgfr1* ^Δ*Frs/*Δ*Frs*^ cochlea (C). (D–F) Hey2 (magenta) is expressed in the prosensory domain (brackets) in sections of E14.5 cochlea (counter-stained with phalloidin: green) (D). Hey2 expression is down-regulated in both *Six1enh21-Cre:: Fgfr1^flox/flox^* mutant cochlea (E) and *Fgfr1* ^Δ*Frs/*Δ*Frs*^ (F). (G–I) Section of control E14.5 cochlea shows Jag1 immuno-labelling in Kölliker's organ. (G). Expression is unchanged in both *Six1enh21-Cre::Fgfr1^flox/flox^* (H) and *Fgfr1* ^Δ*Frs/*Δ*Frs*^ (I). (J–L) In situ hybridization of *Fgf10* on section of E14.5 cochlea shows expression in Kölliker's organ (J). While the expression pattern is unchanged, *Fgf10* expression levels are lower in *Six1enh21-Cre::Fgfr1^flox/flox^* cochlea (K). Expression in *Fgfr1* ^Δ*Frs/*Δ*Frs*^ mutant cochlea is unchanged (L). (M–O) *Bmp4* expression in E14.5 control cochlea is restricted to the outer sulcus (M). The expression pattern is unchanged, however *Bmp4* expression levels are higher in *Six1enh21-Cre::Fgfr1^flox/flox^* cochlea (N). Expression in *Fgfr1* ^Δ*Frs/*Δ*Frs*^ mutant cochlea is unchanged (O). (P) Quantification of relative mRNA level of *Atoh1*, *Hes5*, *p27^Kip1^*, *Hey2*, *Fgf10*, *Jag1* and *Bmp4* in E14.5 cochlear epithelial cells using quantitative PCR. Mean ± SD are shown. *p<0.05. l, lateral; d, dorsal. Scale bar: A–O, 75 µm (in C).

The down-regulation of p27^Kip1^ expression in the prosensory domain indicated that even though prosensory precursors had become post-mitotic, a marker of the prosensory domain was not correctly expressed. Section analysis revealed that as well as p27^Kip1^ (Figure 7A–C), the prosensory domain marker Hey2 was also reduced in cochlea from both *Six1enh21-Cre::Fgfr1^flox/flox^* and *Fgfr1* ^Δ*Frs/*Δ*Frs*^ mutants (Figure 7D–F). We confirmed the down-regulation of *p27^Kip1^* and *Hey2*, as well as two other prosensory markers, *Hes5* and *Atoh1*, by quantitative PCR (Figure 7P). The down-regulation of prosensory domain markers was significantly milder in *Emx2-Cre::Fgfr1^flox/flox^* cochleae than in either *Six1enh21-Cre::Fgfr1^flox/flox^* or *Fgfr1* ^Δ*Frs/*Δ*Frs*^ mutants (Figure 7P).

As well as the prosensory domain, the Sox2-positive sensory patch also forms Kölliker's organ and the outer sulcus. We thus asked if *Fgfr1* mutation also affected these structures. Cells in Kölliker's organ normally express *Fgf10* and Jag1. In both *Six1enh21-Cre::Fgfr1^flox/flox^* and *Fgfr1* ^Δ*Frs/*Δ*Frs*^ mutants, the spatial expression of Jag1 (Figure 7G–I) and *Fgf10* (Figure 7J–L) was unchanged. However, quantitative PCR revealed a down-regulation of *Fgf10* expression, although *Jag1* did not show any significant difference (Figure 7P). The spatial pattern of *Bmp4*, a marker for the outer sulcus located lateral to prosensory domain, was also unchanged in *Six1enh21-Cre::Fgfr1^flox/flox^* and *Fgfr1* ^Δ*Frs/*Δ*Frs*^ mutants (Figure 7M–O). Quantitation revealed up-regulation of *Bmp4* only in *Six1enh21-Cre::Fgfr1^flox/flox^* mutant but not in *Fgfr1* ^Δ*Frs/*Δ*Frs*^ (Figure 7P). These results indicate that although cell cycle exit, an aspect of prosensory domain induction, occured normally, the induction of genes marking the prosensory domain is impaired in the absence of Frs2/3-mediated FGFR1 signalling. This signalling also contributes to the up-regulation of *Fgf10* in Kölliker's organ. However, FGFR1 signalling independently of Frs2/3 recruitment, may negatively regulate *Bmp4* expression in the outer sulcus.

### FGFR1 is necessary for Sox2 maintenance during sensory patch formation

The expression of Sox2 in the sensory patch is known to be critical in the formation of prosensory domain and subsequent HC formation; mutation or reduction in Sox2 expression affects their development in a dose-dependant fashion [2]. Furthermore, FGF signalling has been shown to be sufficient for Sox2 expression [22]. We thus hypothesised that the HC phenotype observed in *Fgfr1* mutants were, in part, due to alterations in Sox2 expression. Initially, Sox2 is expressed in the neuronal and sensory precursors in the otocyst at E10.5. Between E12.5 to E14.5, Sox2 expression in the cochlear duct is detected in the thickened epithelial cells that mark the site of the prosensory domain [32]. By E18.5, Sox2 is confined to the SCs of the organ of Corti [4].

**Figure 8:**
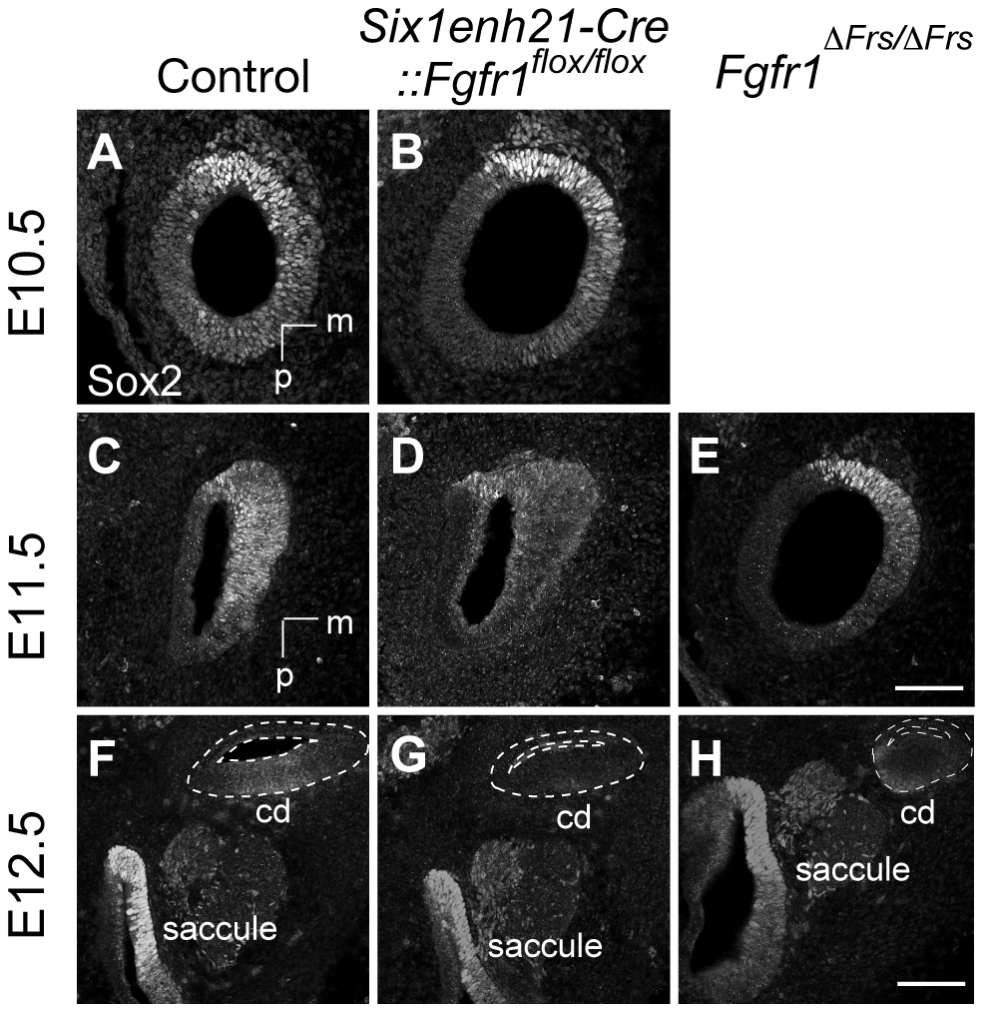
Sox2 is not maintained in FGFR1 signalling mutants. (A, B) Sox2 protein expression in sections of control E10.5 otocyst (A) is comparable to the expression observed in E10.5 *Six1enh21-Cre::Fgfr1^flox/flox^* otocyst (B). (C–E) At E11.5, Sox2 expression is detected in the control otocysts (C), but is down-regulated in *Six1enh21-Cre::Fgfr1^flox/flox^* otocyst sections (D). In *Fgfr1* ^Δ*Frs/*Δ*Frs*^ otocyst, expression is slightly down-regulated, although the morphology of the otocyst is closer to E10.5 (E). (F–H) By E12.5, the cochlear duct expression of Sox2 is apparent in control inner ears (F), however Sox2 is down-regulated in *Six1enh21-Cre::Fgfr1^flox/flox^* mutants (G) and *Fgfr1* ^Δ*Frs/*Δ*Frs*^ inner ears (H) (within dotted lines). By contrast, Sox2 expression in saccular epithelium is unaffected in both mutants. m, medial; p, posterior; cd, cochlear duct. Scale bars: A–E; 75 µm (in E), F–H; 150 µm (in H).

**Figure 9:**
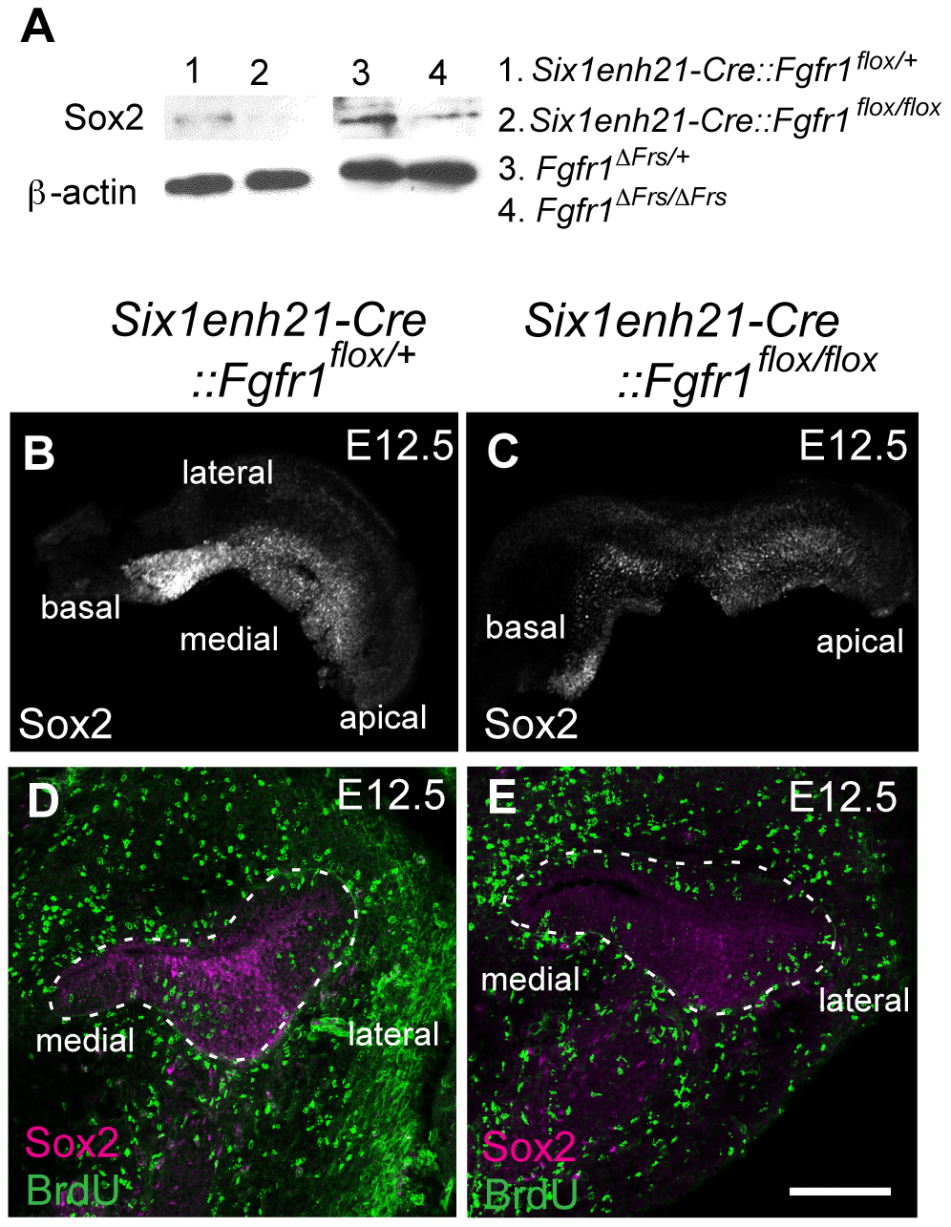
Sox2 is down-regulated prior to prosensory domain formation in FGFR1 signalling mutants. (A) Western blotting of cochlear lysates at E12.5 shows Sox2 protein in heterozygous *Six1enh21-Cre::Fgfr1^flox/+^* (lane 1), mutant *Six1enh21-Cre::Fgfr1^flox/flox^* (lane 2) or heterozygous *Fgfr1* ^Δ*Frs/+*^ (lane 3), and mutant *Fgfr1* ^Δ*Frs/*Δ*Frs*^ (lane 4). Sox2 is decreased in both FGFR1 signalling mutants compared to control. ß-actin is used as a loading control. (B, C) Whole-mount cochlea at E12.5 show normal Sox2 expression in heterozygous controls (B), however Sox2 is down-regulated in *Six1enh21-Cre::Fgfr1^flox/flox^* cochlea (C). (D, E) BrdU-positive cells are present along the width of cochlear duct of control (D) and *Six1enh21-Cre::Fgfr1^flox/flox^* (E) (within dotted lines) although Sox2 immunoreactivity is decreased in the mutant cochlea. Scale bar: D, E, 75 µm (in E).

Sox2 was initially expressed at comparable levels between control, heterozygous, inner ears and *Six1enh21-Cre::Fgfr1^flox/flox^* mutants at E10.5 (Figure 8A and B). By E11.5, expression in *Six1enh21-Cre::Fgfr1^flox/flox^* inner ears was decreased (Figure 8D), although in *Fgfr1* ^Δ*Frs/*Δ*Frs*^ expression levels were equivalent to those in control inner ears (Figure 8C and E). By E12.5, decreased expression of Sox2 in the cochlea of both *Fgfr1* mutant lines was apparent (Figure 8F–H), although Sox2 expression in the saccule was unchanged. To quantify this decrease, we measured Sox2 protein levels in E12.5 mouse cochlea. Levels were reduced by approximately 78% in *Six1enh21-Cre::Fgfr1^flox/flox^* to the levels found in *Six1enh21-Cre::Fgfr1^flox/+^*, while a 55% decrease was observed in *Fgfr1* ^Δ*Frs/*Δ*Frs*^ when compared to heterozygous controls (Figure 9A). This down-regulation was confirmed by immunostaining whole cochleae with Sox2 antibody (Figure 9B and C). To exclude the possibility that the early Sox2 down-regulation occurred due to accelerated prosensory domain development, we used BrdU uptake to indicate its formation. At E12.5, even though Sox2 is down-regulated in the cochlear rudiment of *Six1enh21-Cre::Fgfr1^flox/flox^*, BrdU-positive cells can still be detected (Figure 9D and E), indicating that Sox2 down-regulation occured prior to prosensory domain formation. Furthermore, the down-regulation is not a result of cell survival: No difference in cell death was observed between controls and *Six1enh21-Cre::Fgfr1^flox/flox^* cochleae using an antibody against activated caspase-3 to detect apoptotic cells (data not shown).

**Figure 10:**
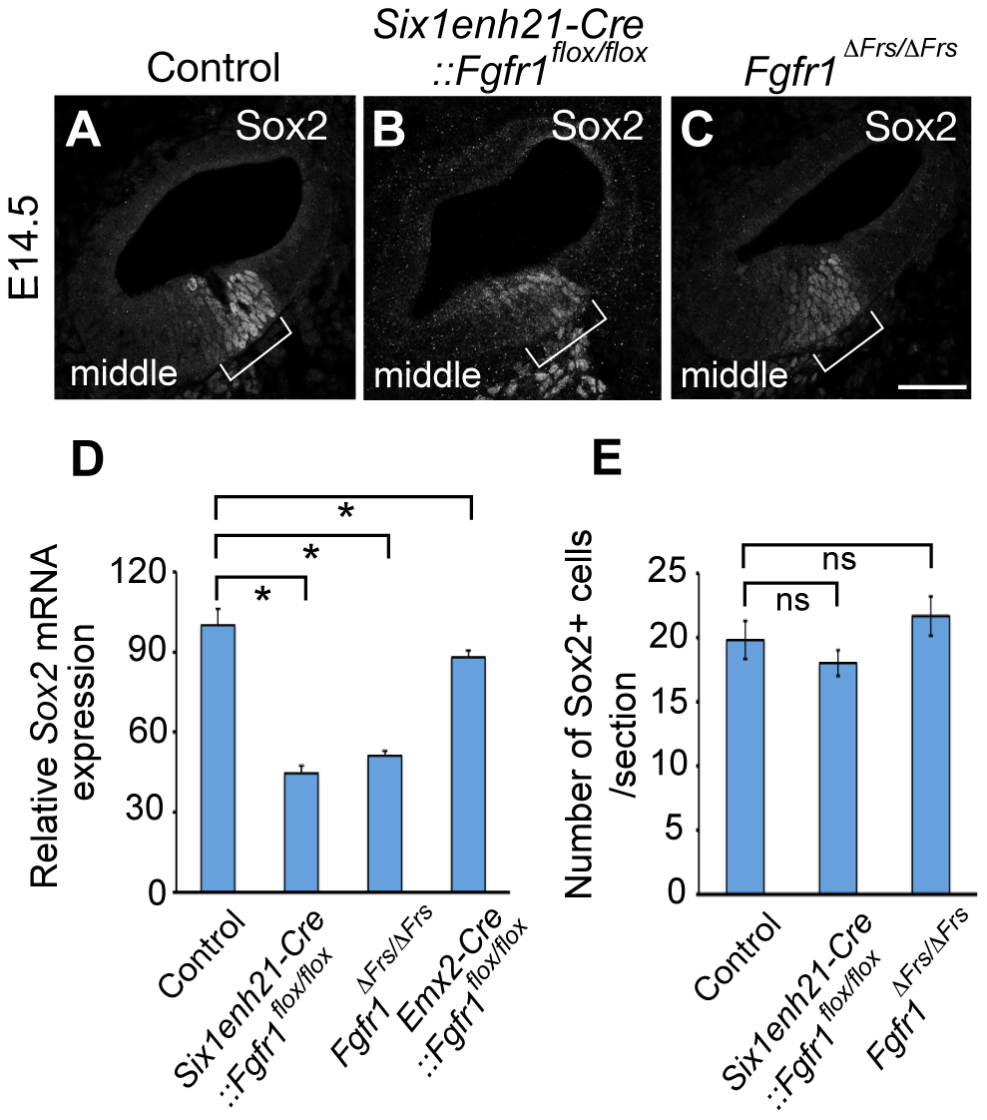
Sox2 is weakly expressed in FGFR1 signalling mutants at the onset of sensory cell differentiation. (A–C) At E14.5, Sox2 expression can be detected in the prosensory domain of control cochlear duct sections (A), but is down-regulated in *Six1enh21-Cre::Fgfr1^flox/flox^* inner ear (B) and *Fgfr1* ^Δ*Frs/*Δ*Frs*^ inner ears (C) (brackets). (D) Quantification of relative mRNA level of *Sox2* in E14.5 cochlear epithelial cells using quantitative PCR. Mean ± SD are shown. *p<0.05. (E) The number of Sox2-positive cells from the middle turn of cochlear duct were counted. Mean ± SD are shown. Scale bar: A–C; 75 µm (in C).

At E14.5, the onset of sensory cell differentiation, Sox2 is expressed robustly in the prosensory domain (Figure 10A). *Six1enh21-Cre::Fgfr1^flox/flox^* (Figure 10B) and *Fgfr1* ^Δ*Frs/*Δ*Frs*^ cochleae (Figure 10C) showed weak Sox2 expression in prosensory domain. When compared to E14.5 heterozygous controls, *Sox2* expression was decreased by approximately 66% in *Six1enh21-Cre::Fgfr1^flox/flox^* mutant cochlea, and by 49% in *Fgfr1* ^Δ*Frs/*Δ*Frs*^. Only a 12% decrease of *Sox2* expression levels was observed in *Emx2-Cre::Fgfr1^flox/flox^* mutants (Figure 10D). To exclude the possibility that reduced *Sox2* expression was as a result of reduced cell numbers, Sox2-positive cells in the prosensory domain were counted (Figure 10E). No significant difference between controls (19.8±1.5: n = 5) and both *Six1enh21-Cre::Fgfr1^flox/flox^* (18±1.0: n = 4) and *Fgfr1* ^Δ*Frs/*Δ*Frs*^ (21.6±1.5: n = 4) cochleae was detected. These results indicate that reduced expression of *Sox2* is independent of cell number. In addition, reduced *Sox2* expression was also detected in SCs of E18.5 *Fgfr1* ^Δ*Frs/*Δ*Frs*^ cochlea (Figure 5G–I).

**Figure 11:**
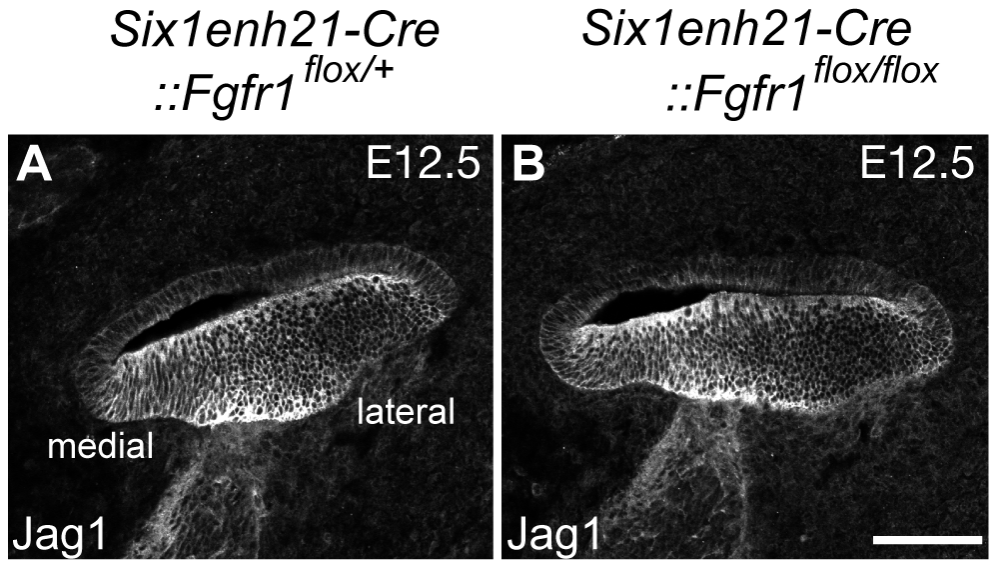
Sox2 down-regulation is independent of Jag1 action. (A, B) Expression of Jag1, the Notch ligand important for Sox2 expression, is unaffected in control (A) and *Six1enh21-Cre::Fgfr1^flox/flox^* cochlea (B) at E12.5. Scale bar: A, B, 75 µm in (B).

Sox2 expression in the sensory patch is induced by activation of the Notch receptor by its ligand Jag1 [6], [14]. Expression analysis of Jag1 in *Six1enh21-Cre::Fgfr1^flox/flox^* mutant revealed that its expression pattern is unchanged (Figure 11A and B), suggesting that FGFR1 signalling affects Sox2 expression independent of any affect on Jag1 regulation. Taken together, we suggest that FGFR1-Frs2/3 signalling is required for Sox2 maintenance in sensory progenitors.

### ERK phosphorylation is repressed in the inner ear of FGFR1 signalling mutants

Frs2/3-mediated FGFR1 signalling is transduced by a number of downstream pathways. We investigated which were activated during Sox2 maintenance in the sensory patch. The MAP kinase pathway is one of the key mediators of receptor tyrosine kinase signalling, and is activated through Frs2/3 recruitment to FGFR1 [25]. To determine if this pathway was activated in the inner ear, we used antibodies specific for the di-phosphorylated form of Erk1 and Erk2 (dpERK), an indicator of MAPK activity [33], to investigate the spatiotemporal activation of this pathway in the inner ear.

**Figure 12:**
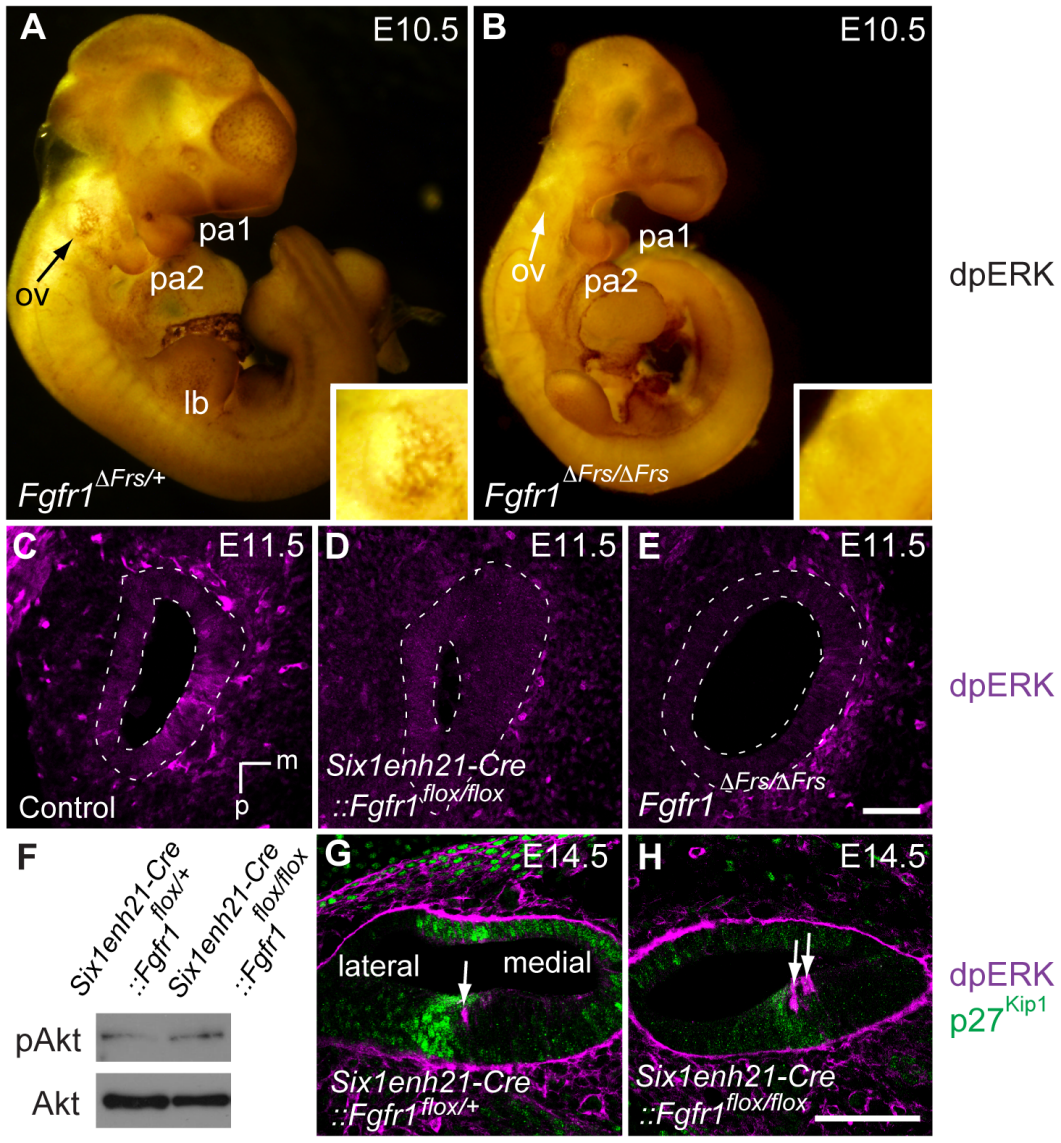
ERK phosphorylation is inhibited in the developing inner ear of FGFR1 signalling mutants. (A, B) Immunostaining for dpERK in E10.5 whole embryos, reveals ERK phosphorylation in the ventral half of the *Fgfr1* ^Δ*Frs/+*^ otic vesicle (arrow, and inset for magnified image) (A). ERK phosphorylation is undetectable in mutant *Fgfr1* ^Δ*Frs/*Δ*Frs*^ otocyst (arrow, and inset for magnified image) (B). For internal control, dpERK localization to rostral edge of PA1 and caudal edge of PA2 is detected in both heterozygotes and homozygotes. (C–E) dpERK immuno-labelling on coronal sections of E11.5 mouse heads. dpERK staining can be detected in the ventromedial wall of the control otocyst (C), but is undetectable in *Six1enh21-Cre::Fgfr1^flox/flox^* (D) and *Fgfr1* ^Δ*Frs/*Δ*Frs*^ otocysts (E) (dashed lines). (F) Western blotting was used to detect phosphorylated and unphosphorylated forms of Akt in protein extracted from E12.5 cochlear epithelia. The level of phosphorylated Akt was unchanged between *Six1enh21-Cre::Fgfr1^flox/+^* and *Six1enh21-Cre::Fgfr1^flox/flox^* cochlea. (G, H) Immuno-labeling of cross sections of the cochlear duct of E14.5 mice with the dpERK and p27^Kip1^ antibodies. In both *Six1enh21-Cre::Fgfr1^flox/+^* (G) and *Six1enh21-Cre::Fgfr1^flox/flox^* (H), the domain of dpERK (magenta) is localized to the medial border of prosensory domain marked by p27^Kip1^ (green) expression (arrows), where nascent IHC and inner pillar cells are present. m, medial; d, dorsal; ov, otic vesicle, pa, pharyngeal arch; lb, limb bud. Scale bars: C–E, 50 µm (in E); G, H, 75 µm (in H).

Our data thus far suggested that FGFR1 activity commencing prior to E12.5 and was necessary for Sox2 maintenance. In agreement with this timing, we detected ventral localization of dpErk in the otocyst of E10.5 *Fgfr1* ^Δ*Frs/+*^ heterozygous embryos (Figure 12A). In contrast, otocyst expression could not be detected in homozygous *Fgfr1* ^Δ*Frs/*Δ*Frs*^ embryos (Figure 12B). At E11.5, sections revealed ventromedial dpErk localization in the otocyst of *Six1enh21-Cre::Fgfr1^flox/+^* heterozygous control (Figure 12C) but is down-regulated in both homozygous *Six1enh21-Cre::Fgfr1^flox/flox^* otocyst as well as *Fgfr1* ^Δ*Frs/*Δ*Frs*^ homozygote embryo (Figure 12D and E). Frs2/3-mediated FGFR1 signalling also activates PI3K, which results in the phosphorylation of Akt [26]. We thus asked if this pathway was also affected in FGFR1 signalling mutants. At E12.5 we found no difference in the levels of phospho-Akt between *Six1enh21-Cre::Fgfr1^flox/+^* heterozygous and *Six1enh21-Cre::Fgfr1^flox/flox^* otocysts (Figure 12F).

At later stages of sensory cell development, FGF8 signalling mediated through FGFR3 is thought to play a role in the specification of pillar and Deiter's cells [34], [35]. To verify the specificity of the FGFR1 signalling mutants, we asked if ERK phosphorylation was affected at these later stages. We found no obvious difference in dpErk localization to the cells of E14.5 *Six1enh21-Cre::Fgfr1^flox/+^* heterozygous and *Six1enh21-Cre::Fgfr1^flox/flox^* homozygous inner ears (Figure 12G and H), where nascent pillar cells IHCs are present. Thus, inhibition of signalling by FGFR1 specifically affects early ERK phosphorylation at E10.5 and E11.5, but does not affect later activation at E14.5 by other FGF receptors.

## Discussion

Formation of cochlear HCs takes place progressively, with the potential of a group of Sox2-positive precursor cells, known as the sensory patch, becoming gradually restricted under the influence of a number of signalling molecules. Our observations suggest that FGFR1 signalling, acting through the adaptor Frs2/3, is responsible for sensory progenitor maintenance, partly through the maintenance of early Sox2 expression, and that in its absence, down-regulation of Sox2 results in a reduction in the number of HCs. However, despite the reduction of early Sox2 expression, subsequent patterning of the sensory patch into the precursor domain of the sensory cells, the prosensory domain, is only partially affected. Surprisingly, even though prosensory domain markers such as p27^Kip1^ and Hey2 are dramatically down-regulated in both the conditional *Six1enh21-Cre::Fgfr1^flox/flox^* and *Fgfr1* ^Δ*Frs/*Δ*Frs*^ allele, a ZNPC is still established normally, and on schedule, showing the normal apical to basal progression. This also implies that the cell cycle inhibitor p27^Kip1^ is required redundantly for sensory progenitors to exit the cell cycle exit. In the mouse, the cell cycle inhibitor p19^Ink4d^ is also found in the sensory progenitors, and is known to act redundantly with p27^Kip1^ [36].

### FGF signalling regulates Sox2 maintenance in the sensory patch

The regulation of Sox2 by FGF signaling has been characterized in a number of other systems, for example during foregut development [37], retinal pigmented epithelia [38], the lens placode [39] and in the differentiation of osteoblasts [40]. We show that in the cochlear precursor, FGF signalling maintains Sox2 expression. The reduction of Sox2 is not as a consequence of reduced proliferation (and hence reduced numbers) of Sox2-positive cells. While the number of proliferating cells in *Six1enh21-Cre::Fgfr1^flox/flox^* cochlea is reduced, the numbers in the *Fgfr1* ^Δ*Frs/*Δ*Frs*^ allele are not. Despite this difference, Sox2 levels are reduced in both mutants at E12.5 and E14.5, suggesting that during cochlear HC formation one role for FGFR1 signaling is in the maintenance of Sox2 expression. Further support for the regulation of Sox2 by FGFR1 signaling comes from the correspondence of HC loss seen in *Six1enh21-Cre::Fgfr1^flox/flox^* and *Fgfr1* ^Δ*Frs/*Δ*Frs*^ cochlea with other mutants. Sensory cell loss is more prominent apically in the cochlea, with the phenotype becoming milder basally. Such phenotypes are similar to knockouts or hypomorphic alleles of *Jag1* and *Sox2* [2], [6], suggesting their involvement in a gene network with *Fgfr1*. Indeed further support for this molecular network comes from explant studies that show that exogenous application of FGF20 can overcome Notch-Jagged-mediated inhibition of Sox2 [22]. One caveat is that it is unclear whether the regulation of Sox2 maintenance by FGFR1 signalling is direct or indirect, through the upstream regulation of other factors important in Sox2 maintenance. It is clear that further studies are necessary to determine the exact mechanism by which FGFR1 signalling regulates Sox2.

At least two roles for Sox2 have been described during the formation of the cochlear sensory cells. The above-mentioned network, apparent from E10.5 to E12.5, maintains the competence of precursor cells to form sensory progenitors. This is supported by the analysis of the cochlear phenotype of mutant mice with little or no Sox2. These mutants show reduced, or absent HCs in the cochlea [2]. A later role for Sox2, from around E15, has been proposed. Here, Sox2 maintains SC fate, and preventing ectopic HC formation through the repression of Atoh1 [4]. This is suggested by hypomorphic alleles where the reduction of Sox2 is not as severe. Here, HC number is increased [2], [4]. Our results suggest that these two activities are separable, with FGFR1 signalling maintaining sensory commitment, partly through Sox2 regulation.

The question remains, how does decreased Sox2 as a result of reduced FGFR1 signalling translate into reduced sensory cells in the cochlea? Sox2 expression as well as other prosensory markers expressed in prosensory domain were down-regulated in both *Six1enh21-Cre::Fgfr1^flox/flox^* and *Fgfr1* ^Δ*Frs/*Δ*Frs*^ mutants, whereas only *Six1enh21-Cre::Fgfr1^flox/flox^* mutants showed defect in cell proliferation. Moreover, both mutants showed similar effects on the formation of HCs. We thus conclude that early cell cycle exit provides, at most, a minor contribution to the disruption of prosensory formation, and hence cochlear HC development in FGFR1 signalling mutants. Instead, it is possible that the level or duration of Sox2 expression determines the commitment or competence to form HCs. A number of studies have described the quantitative requirement for Sox2 in other systems such as in the retinal progenitors [41], anterior foregut [37] and in taste buds [42]. Indeed, over-expression studies have suggested this is also the case in HC [4]. One possible mechanism, through which the duration of Sox2 expression in progenitors and precursors may be translated into effects on commitment and differentiation, is suggested from work on the effects of Sox2 binding to target gene enhancers in other systems [43], [44]. Here silenced genes, important for cell type differentiation, are pre-bound with Sox2. Pre-binding is thought to be associated with the generation of local epigenetic changes [44] or is required for successive binding of co-operative factors [43], important in gene activation, priming the genes for activation. Consistent with this is data showing Sox2 binding sites in the *Atoh1*, a gene that is responsible for sensory cell differentiation in the inner ear [45]. Similarly, we suggest that one function of maintained early Sox2 expression, controlled by FGFR1 signalling, is to prime prosensory genes, such as Atoh1, for subsequent activation and thus control the differentiation of the sensory cells.

The disruption of the transition from Sox2-positive sensory progenitors to prosensory precursors also provides an explanation for the discontinuous “island” phenotype of HCs in the cochlea of FGFR1 signalling mutants. Convergent extension movements that partially drive cochlear extension normally distribute sensory precursors over the length of the organ of Corti [46], . However the fewer numbers of precursors in FGFR1 signalling mutants cannot be evenly dispersed. The apical to basal difference in the distribution of the sensory cells in these mutants may suggest directionality for these rearrangements.

### FGFR1 signalling and inner ear hair cell development

Several studies have proposed FGF20 as the FGFR1 ligand during mouse cochlear development [21], [48]. Indeed there is good correlation of the phenotype between *Fgf20^−/−^* mutants and *Emx2-Cre::Fgfr1^flox/flox^* described in this study; both have moderate reduction in the number of OHC, and IHC remains unaffected. In addition, their prosensory domain formation is largely unaffected. In contrast, there are important differences between *Fgf20* nulls and both *Six1enh21-Cre::Fgfr1^flox/flox^* and *Fgfr1* ^Δ*Frs/*Δ*Frs*^ mutants. In these more severe *Fgfr1* mutants, HC number is more severely reduced and IHC are also affected. Analysis of *Fgf20* nulls revealed a function for Fgf20 in HC differentiation since undifferentiated Sox2-positive cells between sensory islands have been reported [21]. In *Fgfr1* ^Δ*Frs/*Δ*Frs*^ mutant cochleae, however, there are no Sox2-positive cells detected in the lateral compartment among the HC islands. Furthermore, and in contrast to *Fgf20^−/−^* mutant cochlea, Sox2 is down-regulated in both *Six1enh21-Cre::Fgfr1^flox/flox^* and *Fgfr1* ^Δ*Frs/*Δ*Frs*^ mutants from E12.5 to at least E14.5, and prosensory domain formation is disrupted. Our use of the two Cre drivers suggests a reason for this discrepancy. We propose that the FGFR1 has at least two distinct functions in auditory HC development. An early role, prior to E13.5, is in the maintenance of prosensory function, in part through the regulation of Sox2, and in the development of IHC. A later role, in OHC development, is demonstrated by the use of *Emx2-Cre*, which only reaches the same level of driver activity as *Six1enh21-Cre* at E14.5. Here, Sox2 expression in prosensory domain is not severely affected despite significant reduction in OHC numbers. This suggests that a second Fgf ligand, operating either earlier or in combination with Fgf20, is required for the maintenance of Sox2. Although *Fgf20* is expressed in the sensory patches from E10.5 to E14.5 [21], [22], it is likely that prosensory development, but not OHC development, could be compensated by the second ligand in *Fgf20^−/−^* mutant cochlea. A number of Fgf ligands are expressed in the inner ear at these stages of development. *Fgf3*, *-4*, -*5*, *-9*, *-10*, *-16*, as well as *Fgf20* are all detected in the mammalian inner ear at early stages [21], [48]–[54]. Receptor specificity can be used to narrow down the likely early ligand for FGFR1. It is known that mutation of the *Fgfr1-IIIb* isoform does not affect inner ear development, thus it is likely that the *Fgfr1-IIIc* isoform is operating in the sensory epithelium [20]. Of these 7 ligands, FGF4, -5, -9, -16, and FGF20 can bind and signal through FGFR1-IIIc [55], [56], suggesting that one or more of these FGF molecules may act with FGF20 to maintain early *Sox2* expression.

FGF signalling triggers a downstream response, transducing external cues into an internal response. We find that in the absence of Fgfr1, or Frs2/3-mediated FGFR1 signalling, MAP kinase phosphorylation is attenuated, suggesting that this pathway is necessary for sensory progenitor maintenance. The similarity of the *Six1enh21-Cre::Fgfr1^flox/flox^* phenotype with that of *Fgfr1* ^Δ*Frs/*Δ*Frs*^ suggests that adaptor proteins Frs2/3 transduce the FGF signal during sensory progenitor maintenance. However there is an important difference between the two mutants. The defect in proliferation seen in *Six1enh21-Cre::Fgfr1^flox/flox^* (and previously in *Foxg1-Cre::Fgfr1^flox/flox^* [20]) is rescued in *Fgfr1* ^Δ*Frs/*Δ*Frs*^. This suggests the involvement of another downstream pathway in control proliferation in the cochlea. Indeed, the recovery of cell cycle impairment in *Fgfr1* ^Δ*Frs/*Δ*Frs*^ is consistent with previous findings that cell lines obtained from *Fgfr1* ^Δ*Frs/*Δ*Frs*^ are still capable of proliferating [28]. It is likely that other binding partners of FGFR1, such as Grb14, Crk, and Shc, which are known to regulate FGFR1-dependent cell proliferation may respond to mitogenic stimulation in the developing cochlea [57]–[59]. In contrast to the *Fgfr1* ^Δ*Frs/*Δ*Frs*^, which lacks the Frs2/3 interaction motif on FGFR1, mice carrying a point mutation in tyrosine at position 766, *Fgfr1^Y766F/Y766F^* mice, showed no defect in inner ear development. Previous reports have suggested that Y766 phosphorylation may act to negatively regulate FGFR1 activity [30]. It is likely that FGFR1 activity is up-regulated in the inner ear of *Fgfr1^Y766F/Y766F^* mutants. Given that previous studies have suggested that exogenous FGF ligands do not result in an obvious phenotype in the normal mouse cochlea [21], our observation of a normal cochlea in *Fgfr1^Y766F/Y766F^* mice is not unreasonable.

Our analysis of a mutant of *Frs2* in which its subsequent binding to Shp2 is impaired (*Frs2α/2F*) revealed a very early defect in inner ear development, with the inner ear arrested at the otocyst stage (unpublished observations). This phenotype is more reminiscent of the *Fgfr2(IIIb)* mutant, which is thought to mediate signalling from Fgf3 and Fgf10 during inner ear induction [60]. This indicates that Frs2/3-mediated FGFR signalling, like FGF signalling itself, is re-iteratively employed during inner ear formation.

## Materials and Methods

### Ethics statement

Experiments were conducted and mice were housed, in accordance with local (RIKEN CDB) and national guidelines for animal experiments.

### Generation of *Six1enh21-Cre* mice

Full details of the construction of Six1enh21-Cre mice will be presented elsewhere (S. S and K. K., in preparation). Briefly, a transgene was constructed in which the otic/epibranchial progenitor domain (OEPD) enhancer of the Six1 homeobox gene (Six1enh21) [61] was placed upstream of Cre recombinase. Transgenic males were crossed with *Rosa26-flox-STOP-flox-eYFP* reporter females [62] and embryos were collected at stages E8.5 to E11.5, LacZ expression was found in the otic/epibranchial progenitor domain (OEPD) as early as E8.5. At subsequent stages (E9.5 to E11.5), LacZ expression was detected in the otic vesicle and epibranchial placodes/ganglia, scattered cells in the epibranchial ectoderm, the pharyngeal pouch endoderm as well as the olfactory placode/epithelium. The transgenic mouse line, mSix1-21-NLSCre (Acc. No. CDB0466T: http://www.cdb.riken.jp/arg/TG%20mutant%20mice%20list.html), and is available from the RIKEN BioResource Center (BRC).

### Mice

Mice were housed in accordance with local and national guidelines for animal experiments. The *Fgfr1^flox^* mutant mice have been described previously [20]. *Fgfr1^Y766F^* mice were generated by crossing *Fgfr1^n15YF^* with the ubiquitously expressed Cre from *EIIa-Cre* [30]. *Fgfr1^ΔFrs^* mice have been previously described [28]. The *Rosa26-flox-STOP-flox-eYFP* was obtained from Jackson Laboratory (Bar Harbor, ME). The *Atoh1-GFP* line was provided by Dr. Jane Johnson [63]. *Emx2-Cre* mice were provided by Dr. Shinichi Aizawa [64]. *FoxG1-Cre* mice were provided by Jean Herbert, via Carina Hanashima [65]. *Frs2α^2F/2F^* were as described previously [66].

### Immunohistochemistry and histology

Staged mouse heads were fixed in 4% paraformaldehyde for 1–4.5 hours, depending on stage, and then prepared and mounted for cryo-sectioning. Immunofluorescence was performed as has previously been described [67]. The following antibodies were used: anti-p27^Kip1^ (#RB-006-P, Thermo Scientific, Fremont, CA), anti-Sox2 (#AB5603, Millipore, Temecula, CA), anti-pErk1/2 (#4370, Cell Signalling, Beverly, MA), anti-Hey2 (gifted by Neil Segil, House Ear Institute, Los Angeles, USA), anti-Prox1 (#AB5475, Millipore, Temecula, CA), anti-p75 (#AB1554, Millipore, Temecula, CA), anti-BrdU (#555627, BD Pharmingen, Franklin Lakes, NJ), anti- Jag1 (#sc-6011, Santa Cruz Biotechnology, Santa Cruz, CA), anti-GFP (#04406-26. Nacalai Tesque), anti-Caspase-3 (#G748A, Promega, Madison, WI), and anti-Myosin7a (#25-6790, Proteus, Ramona, CA). For BrdU staining, the specimens were pre-treated in 2N HCl for 20 min at 37°C, and neutralized with 0.01M PBS (pH 8.5) for 10 min at room temperature. For whole-embryo dpERK staining, fixed embryos were dehydrated in a graded methanol series and then treated with 5% H_2_O_2_ for 1 hr. Rehydrated embryos were processed as previously described [68]. Signal was detected using DAB substrate kit for peroxidase (Vector). Alexa-488, Alexa-594, or anti-rabbit-HRP (Dako) conjugated secondary antibodies were used to detect primary antibodies. F-actin was detected using phalloidin conjugated to Alexa-488 (Molecular Probes).

### Cell counting and measurement of cochlear length

For cochlear and vestibular HC counting, E16.5–E18.5 samples stained with Myo7a or expressing Atoh1-GFP were used since most *Fgfr1* mutants die before birth. Inner and outer HC were distinguished by location and morphology as described previously [21]. Group of single row of HCs was regarded as IHCs since they were located medial side of p75-expressing pillar cells. Relative cochlear length was measured using ImageJ software. For evaluation of HC number per length, we counted more than 300 µm regions of the base, middle, and apex of the cochlea and normalized counts to 100 µm (more than n = 4 in each HC type) as described [21]. For Sox2-positive cell counting, cross sections from E14.5 samples were made and middle turn of cochlear duct stained with anti-Sox2 antibody was chosen.

### RNA *in situ* hybridization

RNA *in situ* hybridization on cryo-sections was performed as previously described [69].

### Paint-filling

The gross anatomy of bony labyrinths at E14.5 was visualized by paint-filling as previously described [29]. Briefly, decapitated heads were fixed in Bodian's fixative over night. Specimens were subsequently immersed in a graded ethanol series to dehydrate, and cleared in a 2∶1 mixture of benzyl benzoate and benzoic acid (BABB). The inner ears were visualized by injection of 1% white paint in BABB into the common crus.

### Bromo-deoxyuridine incorporation assay

BrdU (100 mg/g body weight) was injected into pregnant mice intra-peritoneally at E10.5–E14.5. BrdU injected mice were sacrificed 2 hours after injection, and then fixed in 4% PFA. BrdU-positive cells were counted in at least four cross sections of the cochlear apical (at E12.5) or middle (at E14.5) turn. For E14.5 samples, only BrdU-labelled cells in Kölliker's organ were counted. Data shown are mean ± SD. P-values were calculated using unpaired t-test, to determine the significance of the difference between experimental and control samples.

### Quantitative reverse transcription PCR

Whole otocyst or cochlear epithelial cells were dissected from embryos at E10.5–E14.5 (at least n = 2 in each sample). Enzymatic treatment was conducted to remove mesenchyme [70]. Total RNA from pure otic epithelial cells was extracted using the RNAqueous-Micro kit (#AM1931, Ambion, Austin, TX) and then reverse-transcribed using First Strand cDNA Synthesis Kit for RT-PCR (#11483188001, Roche, Indianapolis, IN). Synthesized cDNA and primer sets were mixed with Power SYBR Green PCR Master Mix (#4367669, Applied Biosystems, Warrington, UK), and real-time quantitative PCR was performed using an ABI Prism 7900 Sequence Detection System (Applied Biosystems). All reactions were carried out in duplicate. The relative amount of mRNA was calculated by standard curve method, and normalized to that of 36B4 mRNA [71]. P-values were calculated using unpaired t-test, to determine the significance of the difference between experimental and control samples.

### Immunoblotting

E12.5 or E14.5 cochlear epithelial cells, purified from underlying mesenchymal cells were lysed in a buffered solution, consisting of SDS, salt, phosphatase inhibitor, and proteinase inhibitor. A mixture of lysate, sample buffer, and 2-mercaptoethanol, were boiled at 98°C for 2 min and separated on a SuperSep Ace gel (Wako), and subsequently transferred into PVDF membrane (GE Healthcare). The following antibodies were used: rabbit anti-Akt antibody (1∶ 1000) (#9272, Cell Signalling Technology), anti-Sox2 (1∶ 1000) (#AB5603, Millipore, Temecula, CA), rabbit anti-Phospho Akt antibody (1∶ 1000) (#9271, Cell Signalling Technology), and anti-Actin antibody (1∶ 10000) (MBL). Horseradish peroxidase-linked anti-rabbit IgGs were used as secondary antibodies (1∶10,000) (GE Healthcare) and revealed using Amersham ECL Prime Western Blotting Detection Reagent (GE Healthcare) according to the manufacturer's instruction. ImageJ software was used to compare the relative Sox2 protein amount between control and *Fgfr1* mutants.
